# When Elon Musk Changes his Tone, Does Bitcoin Adjust Its Tune?

**DOI:** 10.1007/s10614-021-10230-6

**Published:** 2022-01-13

**Authors:** Toan Luu Duc Huynh

**Affiliations:** 1 UEH Institute of Innovation (UII), University of Economics Ho Chi Minh City (UEH), 59C Nguyen Dinh Chieu Street, District 3, Ho Chi Minh, Vietnam 70000 Vietnam; 2 Chair of Behavioral Finance, WHU - Otto Beisheim School of Management, Burgplatz 2, 56179, Vallendar, Germany

**Keywords:** Bitcoin, Elon Musk twitter, Negative, Positive, Optimistic, Pessimistic, C22, G15

## Abstract

We present a textual analysis that explains how Elon Musk’s sentiments in his Twitter content correlates with price and volatility in the Bitcoin market using the dynamic conditional correlation-generalized autoregressive conditional heteroscedasticity model, allowing less sensitive to window size than traditional models. After examining 10,850 tweets containing 157,378 words posted from December 2017 to May 2021 and rigorously controlling other determinants, we found that the tone of the world’s wealthiest person can drive the Bitcoin market, having a Granger causal relation with returns. In addition, Musk is likely to use positive words in his tweets, and reversal effects exist in the relationship between Bitcoin prices and the optimism presented by Tesla’s CEO. However, we did not find evidence to support linkage between Musk’s sentiments and Bitcoin volatility. Our results are also robust when using a different cryptocurrency, i.e., Ether this paper extends the existing literature about the mechanisms of social media content generated by influential accounts on the Bitcoin market.

## Introduction

Currently, Elon Musk is the wealthiest person in the world (Klebnikov, 2021); however, this information is not surprising enough in the cryptocurrency market. Investors started following Musk’s Twitter account in 2021 when he began tweeting about Bitcoin and providing financial advice. Musk’s Twitter account has more than 55.7 million followers. Noticeably, the value of Bitcoin and most cryptocurrencies skyrocketed at the beginning of February 2021 when Tesla’s CEO, Elon Musk, announced on Twitter that his company spent 1.5 billion USD to buy Bitcoin. This is only one of thousands of potentially market influencing tweets, primarily tweets about cryptocurrency and the equity market that Musk has shared. After Musk’s endorsement, the phenomenon associated with his tweets exemplifies a *signal advance.* Previous studies have explored how Twitter can impact the cryptocurrency market (Kraaijeveld & De Smedt, 2020; Naeem et al., 2020a, 2020b; Öztürk & Bilgiç, 2021; Shen et al., 2019). However, investigations into the effects Musk may have on this market have not been conducted. Recently, Huynh (2021) investigated whether Donald Trump’s Twitter content, which is considered an aggregate market risk with positive attitudes, could drive the Bitcoin market. Differing from Trump’s tweets, remembering that Trump was President of the United States at the time, Musk’s tweets include some jest content. Nevertheless, the market still moves substantially. Thus, regardless of whether Musk’s tweets are perceived as financial advice or recommendations, investors are likely to follow the implied messages to obtain *advantageous information* in cryptocurrency market trading. Acknowledging the growing momentum of financial contagion literature (Akhtaruzzaman et al., 2021a, 2021b; Boubaker & Jouini, 2014), this paper is positioned to explain the role of social media on financial literature. We assume that the market may undergo an (un)expected shake-up due to the release of news. This effect might be stronger due to the speed at which such information is shared. This study attempts to explain the effects of social media on the cryptocurrency market with a focus on the financial contagion perspective (Corbet et al., 2020).

Elon Musk said, “*I always have optimism, but I'm realistic. It was not with the expectation of great success that I started Tesla or SpaceX*” (Kidder, 2012). Motivated by this statement, we hypothesize that Musk tends to express positive attitudes on social media platforms. Therefore, the market may pay attention to his optimistic voice, and his sentiments can drive trading behavior. A previous study (Ante, 2021) provided some empirical evidence that Musk's Twitter content could affect the cryptocurrency market. However, this was an event study that classified the distinguishing feature of his referral content. Therefore, rather than an event study, we perform systematic analyses of Musk’s sentiments using textual analysis. Various empirical evidence indicates that different tones are associated with different economic behaviors, e.g., M&A deals (Wu et al., 2021), risk-taking behavior (Del Gaudio et al., 2020), and financial activities (Druz et al., 2020). Previous textual analysis studies have explored various aspects, e.g., report readability and liquidity (Boubaker et al., 2019), as well as news diversity and market crashes (Boubaker et al., 2021). However, in the cryptocurrency market context, this matter of sentiment remains unanswered. Thus, this study attempts to answer three main research questions.

- (RQ1) What is Elon Musk’s tone?
- (RQ2) Can his tone predict Bitcoin markets?
- (RQ3) Is there a time-varying relationship between tone and the Bitcoin market?

*How does this paper present the novelty in crisis and risk management literature?* Due to the growing momentum of technological developments, financial markets are becoming increasingly volatile due to information leaks in social media, e.g., Trump’s tweets and stock market prices (Burggraf et al., 2020), and COVID-19 news (Ambros et al., 2020; Huynh et al., 2020a, 2020b). Although the current literature has examined the role of presidential Twitter content, e.g., Donald Trump in Huynh (2021), empirical evidence does not explain how an influential person can drive the market. Existing studies have investigated the number of tweets or specific content; however, few studies provide sentiment analysis. Therefore, this study can clarify two current puzzles. First, we examine the potential risk exposure raised by Musk’s Twitter account, which has 55.7 million followers. Second, by employing a cutting-edge method with different dictionary sources, the study explicitly demonstrates the dynamics of his tone (positive, negative). Then, using these proxies of risk, we test the spillover effects and the predictive power on cryptocurrency returns and volatilities. The cryptocurrency market is sensitive to social media news (Li et al., 2021); thus, our paper looks at the new market, particularly cryptocurrency market, and also contributes to recent developments in computational economics. In particular, this paper combines textual analysis with a newly updated dictionary and an advanced econometrics model.

In this study, we refer to the theoretical frameworks presented by Schwert (1981), Wagner et al. (2018), and Huynh (2021). These studies emphasized how the news could be associated with financial asset price changes. This association means that, if the market has the optimal equilibrium with information, any changes, positive or negative, would require the expected discounted payoff to shift to the previous equilibrium. We assume that good news from Musk could positively predict higher returns and vice versa. However, the characteristics of Bitcoin as an alternative investment or hedging instrument are inconclusive (Urquhart & Zhang, 2019; Yuneline, 2019). Thus, some investors might think Bitcoin is a better safe-haven for uncertainties raised by Musk. Therefore, our study will benefit from the previous theoretical frameworks to examine how the newly raising Twitter account could predict Bitcoin price changes. Several studies have presented empirical evidence demonstrating the relationship between the sentiment expressed in tweets and financial markets, e.g., the happiness sentiment (Kraaijeveld & De Smedt, 2020; Li et al., 2017; Sun et al., 2016; Zhao, 2020). With reference to the cryptocurrency market, Shen et al. (2019) concluded that Twitter significantly influences Bitcoin variance. Similarly, Kraaijeveld and Smedt (2020) employ textual analysis to investigate whether Twitter bots could predict market activity in a wide range of cryptocurrencies, including Bitcoin, Litecoin, and Bitcoin-Cash. The findings exhibit the prediction and causality, which highlights the utmost importance of sentiments on this market. We use a large dataset comprised of Elon Musk tweets (10,850 tweets with 157,378 words) posted from December 2017 to May 2021. We performed the textual analysis by counting the positive and negative words found in three financial dictionaries developed by Loughran and McDonald (2011), Bodnaruk et al. (2015). As a result, we identify Elon Musk’s sentimental indicators for optimistic (or positive) and pessimistic (or negative) tones. The detailed calculation will be discussed in the Sect. 2. Then, the dynamic conditional correlation–generalized autoregressive conditional heteroscedasticity (DCC-GARCH) model devised by Engle (2002) is employed to examine the time-varying connectedness before analyzing the short-run shocks of tones on Bitcoin returns and volatility, estimated by Threshold-GARCH, (T-GARCH). The DCC-GARCH model was selected because it does not employ an arbitrarily selected window size in order to retrieve dynamic connectedness measures.

Our key findings are as follows. First, a positive tone dominates Musk’s tweets, which implies that he tends to use optimistic words to express his ideas in his Twitter account. Second, Musk's tone has predictive power relative to Bitcoin returns. In addition, after rigorously controlling other determinants, we found a causal relationship for both positive and negative tone on Bitcoin returns. Concomitantly, there exist the reversal effects on the positive tone when the signs of coefficients change. Third, there is no significant relationship between Musk's tone and Bitcoin volatility. Forth, we also observed time-varying connectedness between Musk’s attitudes and Bitcoin markets over a four-year period (2017–2021). In addition, the average effects of the optimistic tone are stronger than the pessimistic tone.

Compared with existing literature that examines the relationship between DOGE and DOGE’s father (Cary, 2021), this is the first study to investigate the impacts of Musk’s sentiments on Bitcoin and Ethereum returns and volatility. While previous studies focus on specific events (Ante, 2021) and the frequency of posting tweets (Tandon et al., 2021), this study employed existing financial dictionaries (Loughran and McDonald (2011), Bodnaruk et al. (2015) to analyze the sentimental proxies. Hassan et al. (2021) collected 15,000 tweets about cryptocurrency and evaluated eight emotions reflected in those tweets; however, they did not analyze their impacts on the cryptocurrency market. Similarly, only one study explores the predictive power of Donald Trump’s tweets on Bitcoin prices, volatility, and trading volumes (Huynh, 2021). Here, it is important to note that, as President of the United States, Donald Trump may be a more influential person than Elon Musk, who, although extremely wealthy, is originally from the business world. The predictive power of a businessman’s tweets on the cryptocurrency market has not yet been examined. From this starting point, we employed cutting-edge econometrics, such as connectedness from the two-step DCC-GARCH model.

This study contributes to the existing literature in three ways. First, we provide empirical evidence that posts from an influential individual on a specific social media platform, i.e., Twitter, can drive investor sentiment and disrupt the cryptocurrency market. Attentive investors are likely to follow financial and trading advice from the social media influencer. Thus, our study sheds new light on the analyses of sentiment in tweets, i.e., positive (optimistic) and negative (pessimistic) views on Bitcoin markets from a well-known billionaire. Second, we document the characteristics of the Bitcoin market. This market is likely to be fragile in response to sudden news. Investors demonstrate irrational behavior and tend to follow to bullish or bearish sentiment when an influential person tweets about the market. Thus, this market can easily be manipulated and distorted by a single social media account. Third, while the effects on Bitcoin returns are evident, we do not find any supporting evidence for Bitcoin volatility. This confirms that *noise news* from Elon Musk sentiments are associated with Bitcoin prices in the short run. However, there is no relationship with volatility in both the short and long term. Our findings indicate that, relative to reacting to news, the Bitcoin market is efficient and immediately regains market equilibrium. Therefore, we conclude the Elon Musk’s effects could not persist in the long term.

The remainder of this paper is organized as follows. Section 2 summarizes the data and methodology used throughout this paper. Empirical results are reported in Sect. 3, and conclusions and policy implications are presented in Sect. 4.

## Data and Methodology

### Data

We retrieved data from Refinitiv to identify necessary variables, such as Bitcoin prices (Bitstamp), gold prices (London Bullion Market), platinum prices (London Platinum and Palladium Market), the US Credit Default Swaps (CDS) premium index, and the FTSE global bond index. We calculate the natural logarithm daily return for Bitcoin, CDS, and global bonds. Note that Bitcoin data collected following the approach proposed by Alexander and Dakos (2020). The ratio of Gold and Platinum prices was calculated using the approach proposed in a study by Huynh et al. (2020a, 2020b). Table 1 summarizes the descriptive statistics of our main variables. Our daily data covers the period from December 2017 to May 2021. We chose this period is to match the timeframe of Musk’s collected tweets. Elon Musk, the Doge’s father, has actively used his social media account since December 2017. In addition, the research period should end prior to the big crash of the cryptocurrency market (May 2021) (Min et al., 2021).

**Table 1:** Summary of descriptive statistics

	BITCOIN	GP	CDS	BOND	RAI	NEGSENT	POSSENT
Mean	0.005	1.724	− 0.002	0.000004	26.153	0.016	0.018
Variance	0.002	0.087	0.003	0.000036	220.742	0.00037	0.00082
Skewness	0.067	0.748***	− 6.388***	− 0.999***	0.169	2.609***	1.993***
	(0.605)	(0.000)	(0.000)	(0.000)	− 0.192	(0.000)	(0.000)
Kurtosis	3.070***	− 0.886***	108.856***	17.405***	− 1.166***	10.928***	6.979***
	(0.000)	(0.000)	(0.000)	(0.000)	(0.000)	(0.000)	(0.000)
JB	136.559***	43.731***	173,685.794***	4437.732***	21.293***	2120.491***	934.057***
	(0.000)	(0.000)	(0.000)	(0.000)	(0.000)	(0.000)	(0.000)
ERS	− 5.039***	− 1.091	− 10.972***	− 6.789***	− 1.943*	− 5.565***	− 6.538***
	(0.000)	(− 0.276)	(0.000)	(0.000)	(− 0.053)	(0.000)	(0.000)
Q^2^ (20)	11.169**	1717.279***	2.696	19.474***	1110.135***	4.923	6.797
	(− 0.04)	(0.000)	(− 0.861)	(0.000)	(0.000)	(− 0.518)	(− 0.274)
LiMak(20)	11.649**	1699.203***	0.141	154.637***	1117.187***	2.485	6.994
	− 0.032	(0.000)	(− 1.000)	(0.000)	(0.000)	(− 0.887)	(− 0.254)

The row of skewness refers to the test of D'Agostino (1970) while kurtosis refers to the test of Anscombe and Glynn (1983). The symbol ‘JB’ denotes Jarque and Bera (1980) for testing normality of distribution. The following tests including ERS, Q2(20), and LiMak (20) are unit-root test and two weighted portmanteau tests, respectively. The numbers in brackets are reported in *p*-value

*< 0.1; **< 0.05; ***< 0.01

We collected all 10,850 tweets posted to Elon Musk’s account (*@elonmusk*) from December 2017 to May 2021. As mentioned previously, we used the dictionaries developed by Loughran and McDonald (2011) and Bodnaruk et al. (2015) to count the number of positive and negative words in Musk’s tweets. We also used the financial dictionary developed by to enhance the understanding of the psychological expressions posted by the most influential account in the market. We counted specific sentiment words in financial contexts, e.g., *loss(es), impairment,* and *adverse(ly)*) and followed the methodology proposed by Huynh (2021) to calculate negativity as the proportion of the number of negative words over the total words. This concept holds for positivity. The concept is widely accepted in financial studies, e.g., mergers and acquisitions (Katsafados et al., 2021), and cryptocurrency and Twitter (Huynh, 2021). Therefore, the concept implies a potential direction for economics and finance studies (Gogas & Papadimitriou, 2021). Differing from the Thomson Reuters News Analytics (TRNA) system and the Thomson Reuters MarketPsych Index (TRMI), our study started with raw data extracted from specific Elon Musk tweets. In other words, we focus on a particular individual rather than using aggregated market data (TRNA and TRMI) to clarify the impacts of an influential social media account on the cryptocurrency market. Then, we performed textual analysis to calculate positivity and negativity before analyzing predictive models.

Overall, all variables exhibit abnormal distribution and are stationary based on the unit root test developed by Elliott et al. (1996). Moreover, all variables show ARCH errors at a 1% significance level, suggesting that using a multivariate GARCH procedure could be a sensible approach. Bitcoin showed positive returns over this period (December 2017 to May 2021), and Musk is inclined to use a positive tone rather than a negative tone when tweeting, which implies that the effects of a positive tone could have stronger effects on the Bitcoin market.

### Methodology

In this study, we use the two-step DCC-GARCH model. The conventional DCC-GARCH (1,1) process is expressed as follows.

1 $$ {\text{y}}_{{\text{t}}} = \mu_{{\text{t}}} + \varepsilon_{{\text{t}}} \;\varepsilon_{{\text{t}}} |{\text{F}}_{{{\text{t}} - 1}} \sim {\text{N}}\left( {0,{\text{H}}_{{\text{t}}} } \right) $$

2 $$ {\upvarepsilon } = H_{t}^{\frac{1}{2}} u_{t} \;u_{t} \sim {\text{N}}\left( {0,I} \right) $$

3 $$ {\text{H}}_{{\text{t}}} = {\text{D}}_{{\text{t}}} {\text{R}}_{{\text{t}}} {\text{D}}_{{\text{t}}} $$

Here, ${\text{F}}_{{{\text{t}} - 1}}$ represents all relevant information available at time (t − 1). We also observe an ${\text{N}} \times 1$ matrix where ${\text{y}}_{{\text{t}}}$, $\mu_{{\text{t}}}$, $\varepsilon_{{\text{t}}}$, and $u_{t}$ represent the mean value in the main time-series variable, a conditional regime, the error term for Eq. 1, and a standardized error term, respectively. To be more precise, Eq. 3 has ${\text{N}} \times {\text{N}}$ matrices, ${\text{R}}_{{\text{t}}}$, ${\text{H}}_{{\text{t}}}$, and ${\text{D}}_{{\text{t}}}$, which represent the dynamic conditional correlations, matrices for time-varying conditional variance–covariance, and variances in time-varying conditional status, respectively. Note that ${\text{D}}_{{\text{t}}}$ is defined as ${\text{D}}_{{\text{t}}} = {\text{diag}}\left( {h_{11t}^{1/2} , \ldots ,h_{NNt}^{1/2} } \right)$.

Initially, we followed previous studies (Bollerslev, 1986; Hansen & Lunde, 2005) for GARCH estimations (Eq. 4) to generate one shock and one persistency parameters. Then, we can see that

4 $$ {\text{h}}_{{{\text{ii}},{\text{t}}}} = \omega + \alpha \varepsilon_{{{\text{t}} - 1}}^{2} + \beta {\text{h}}_{{{\text{ii}},{\text{t}} - 1}} $$

where $h_{t}$ denotes the estimated shock for time t. In addition, $\omega$, $\alpha ,$ and $\beta$ are parameters of constant term, squared error term, and the previous term of estimated shock, respectively. Then, the dynamic conditional correlation is calculated as follows.

5 $$ {\text{R}}_{{\text{t}}} = {\text{diag}}\left( {{\text{q}}_{{{\text{iit}}}}^{{ - 1/2{ }}} , \ldots ,{\text{ q}}_{{{\text{NNt}}}}^{{ - 1/2{ }}} } \right){\text{ Q}}_{{\text{t}}} {\text{ diag}}\left( {{\text{q}}_{{{\text{iit}}}}^{{ - 1/2{ }}} , \ldots ,{\text{ q}}_{{{\text{NNt}}}}^{{ - 1/2{ }}} } \right) $$

Finally, estimations for conditional ($$Q_{t} ) $$ and unconditional standardized $$\left( {\overline{{Q_{t} }} } \right)$$ residual variance–covariance matrices are represented with $${\text{N}} \times {\text{N}}$$ dimensions.

6 $$ {\text{Q}}_{{\text{t}}} = \left( {1 - {\text{a}} - \beta } \right){\text{Q}}_{{\text{t}}} + {\text{au}}_{{{\text{t}} - 1}} {\text{u}}_{{{\text{t}} - 1}}^{^{\prime}} + {\text{bQ}}_{{{\text{t}} - 1}} $$

Here, $a\left( \alpha \right)$ and $b\left( \beta \right)$ represent shocks having nonnegative values and parameters having persistency. It is important to fully satisfy the condition $a + b < 1 \left( {\alpha + \beta \le 1} \right)$. If the condition is satisfied, we can obtain the time-varying parameters ${\text{Q}}_{{\text{t}}}$ and ${\text{R}}_{{\text{t}}}$. If the condition is not satisfied, this process returns the constant conditional correlation (CCC-GARCH) (Bollerslev, 1990). After estimating the DCC-GARCH, we employ the generalized impulse response functions (GIRF) technique (Koop et al. 1996; Pesaran & Shin, 1998). This approach relies on obtaining an independent variable with J-step-ahead impact of variable i on variable j. The generalized process can be written as follows.

7 $$ {\text{GIRF}} = \left( {{\text{J}},\delta_{{{\text{j}},{\text{t}}}} ,{\text{F}}_{{{\text{t}} - 1}} } \right) = {\text{E}}\left( {{\text{y}}_{{{\text{t}} + {\text{J}}}} {|}\varepsilon_{{{\text{j}},{\text{t}}}} = \delta_{{{\text{j}},{\text{t}}}} ,{\text{F}}_{{{\text{t}} - 1}} } \right) - {\text{E}}\left( {{\text{y}}_{{{\text{t}} + {\text{J}}}} {|}\varepsilon_{{{\text{j}},{\text{t}}}} = 0,{\text{F}}_{{{\text{t}} - 1}} } \right) $$

To estimate the volatility impulse response function (VIRF), we rely on the conceptual framework of Eq. 7 where $\delta_{{{\text{j}},{\text{t}}}}$ is a selection vector with a one at the j^th^ position and zero otherwise. A detailed description of a generalized function with time-varying conditional volatility can be found in the literature (Gabauer, 2020). Following Gabauer (2020), the generalized function can be expressed as follows.

8 $$ \Psi^{g} = {\text{VIRF}}\left( {{\text{J}},\delta_{{{\text{j}},{\text{t}}}} ,{\text{F}}_{{{\text{t}} - 1}} } \right) = {\text{E}}\left( {{\text{H}}_{{{\text{t}} + {\text{J}}}} {|}\varepsilon_{{{\text{j}},{\text{t}}}} = \delta_{{{\text{j}},{\text{t}}}} ,{\text{F}}_{{{\text{t}} - 1}} } \right) - {\text{E}}\left( {{\text{H}}_{{{\text{t}} + {\text{J}}}} {|}\varepsilon_{{{\text{j}},{\text{t}}}} = 0,{\text{F}}_{{{\text{t}} - 1}} } \right) $$

After extracting the VIRF, we selected the method proposed by Diebold and Yilmaz (2012, 2014) to compute how the variance of one variable can be explained by other variables. This method is called generalized forecast error variance decomposition. Similarly, we also generalized and normalized the variance such that all numbers in a row will add up to one.

9 $$ \tilde{\phi }_{{{\text{ij}},{\text{t}}}}^{{\text{g}}} \left( {\text{J}} \right) = \frac{{\mathop \sum \nolimits_{{{\text{t}} = 1}}^{{{\text{J}} - 1}} {\Psi }_{{{\text{i}},{\text{j}},{\text{t}}}}^{{2,{\text{g}}}} }}{{\mathop \sum \nolimits_{{{\text{j}} = 1}}^{{\text{N}}} \mathop \sum \nolimits_{{{\text{t}} = 1}}^{{{\text{J}} - 1}} {\Psi }_{{{\text{i}},{\text{j}},{\text{t}}}}^{{2,{\text{g}}}} }} $$

Here, $\mathop \sum \limits_{{{\text{t}} = 1}}^{{{\text{J}} - 1}} \Psi_{{{\text{i}},{\text{j}},{\text{t}}}}^{{2,{\text{g}}}}$ explains the cumulative effect of the *j*th shock and $\mathop \sum \limits_{{{\text{j}} = 1}}^{{\text{N}}} \mathop \sum \limits_{{{\text{t}} = 1}}^{{{\text{J}} - 1}} \Psi_{{{\text{i}},{\text{j}},{\text{t}}}}^{{2,{\text{g}}}}$ represents the aggregate cumulative of all shocks that can be determined from the variance of the analyzed variance. It is important to observe two conditions from Eq. 9 as follows.

10 $$ \begin{array}{*{20}c} {\mathop \sum \limits_{{{\text{j}} = 1}}^{{\text{N}}} \tilde{\phi }_{{{\text{ij}},{\text{t}}}}^{{\text{g}}} \left( {\text{J}} \right) = 1} \\ {\mathop \sum \limits_{{{\text{i}},{\text{j}} = 1}}^{{\text{N}}} \tilde{\phi }_{{{\text{ij}},{\text{t}}}}^{{\text{g}}} \left( {\text{J}} \right) = {\text{N}}} \\ \end{array} $$

From this starting point, we can use CFED to construct the total connectedness index $\left( {{\text{C}}_{{\text{t}}}^{{\text{g}}} \left( {\text{J}} \right)} \right)$.

11 $$ {\text{C}}_{{\text{t}}}^{{\text{g}}} \left( {\text{J}} \right) = \frac{{\mathop \sum \nolimits_{{{\text{i}},{\text{j}} = 1,{\text{i}} \ne {\text{j}}}}^{{\text{N}}} \tilde{\phi }_{{{\text{ij}},{\text{t}}}}^{{\text{g}}} \left( {\text{J}} \right)}}{{\text{N}}} $$

The *total directional connectedness TO others, total directional connectedness FROM others,* and *net total directional connectedness* are measured based on the approach proposed by Diebold and Yilmaz (2012, 2014). We also estimate *net pairwise directional connectedness* (NPDC) between variable i and variable j as follows.

12 $$ {\text{NPDC}}_{{{\text{ij}}}} \left( {\text{J}} \right) = \phi_{{{\text{ji}},{\text{t}}}}^{{\text{g}}} \left( {\text{J}} \right) - \phi_{{{\text{ij}},{\text{t}}}}^{{\text{g}}} \left( {\text{J}} \right) $$

After calculating this index, a positive value of NPDC (variable i) implies the dominating position while a negative value means the dominated position by variable j.

After testing the time-varying connectedness, we employed vector autoregressive models to examine the short-term effects of Musk’s sentiments on Bitcoin returns and volatility. Furthermore, by employing this approach, we can examine whether Musk sentiments are causally associated with the Bitcoin market. In the specific Sect. 3, we generalize the model specifications and describe the execution of empirical testing.

## Findings and Results

### Dominant Position of Positive Attitudes by Elon Musk

In this section, we use the two-sample t-test with equal variances to determine positive and negative sentiments in Musk’s tweets. The means of optimistic and pessimistic sentiments are 0.0228 and 0.0190, respectively (*p* < 0.05). This implies that the average number of positive attitudes are greater than the average number of negative attitudes over the target four-year period. The results are robust when using the paired t-test (*p*-value < 0.01). We found that the correlation between the two indicators was 0.3439, implying significant number of positive tweets at a 1% significance level. Therefore, we can conclude that Elon Musk is likely to use positive words when expressing his attitudes.

### Dynamic Connectedness Across Bitcoin Returns and Elon Musk’s Sentiments

**Fig. 1:**
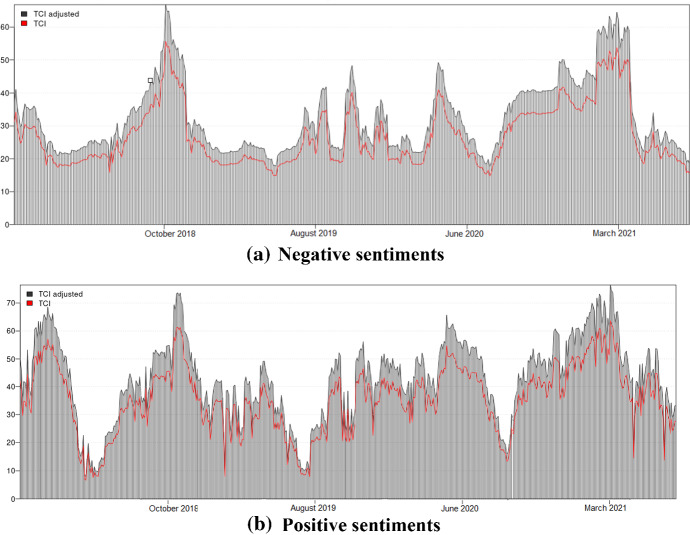
Total dynamic connectedness between Bitcoin returns and Elon Musk’s sentiments. *Notes*: The total dynamic connectedness between Bitcoin returns and each Elon Musk tone (positive and negative attitudes) by using the Dynamic Conditional Correlation–Generalized Autoregressive Conditional Heteroscedasticity (DCC-GARCH) models. Our research period is from December 2017 to May 2021. TCI denotes the total connectedness index. The figure practically implies that the connectedness between Elon Musk’s sentiment and Bitcoin returns

Figure 1 represents the total dynamic connectedness between Elon Musk’s tone and Bitcoin returns. Even when all control variables remain unchanged, we expected positive sentiments to show a higher spillover effect than negative sentiments Comparing two regimes, we can observe that the market’s sensitivity to words conveys a good sense of positivity. Furthermore, both sub-figures depict the dynamic total connectedness indices spanning approximately 40% and 70%, suggesting substantial effect and time-varying changes. There are two periods worth mentioning, i.e., October 2018 and March 2021. The former experienced a peak prior to the Bitcoin crash, and the latter experienced spikes of Bitcoin prices to a new price level (more than $60,000 USD in 2021). Thus, it can be concluded that Bitcoin markets tend to be sensitive to news when prices reach a high level, suggesting a strong relationship between sentiments and Bitcoin returns. Our study confirmed previous empirical evidence that Musk’s Twitter feed only drives trading activities in the short-term period (Ante, 2021). We also found that the tone of his tweets could disrupt the cryptocurrency market.

Looking at closer pairwise connectedness at the averaged dynamic connectedness, we found that the static total connectedness in the positive domain (35.46%) is higher than the negative domain (27.04%) (Appendix Tables 5 and 6). Therefore, we can conclude that when using DCC-GARCH estimations for connectedness, there is a time-varying spillover between the tone of an influential Twitter account and the Bitcoin market. Our findings are also consistent with previous studies about the influence of Trump's Twitter account on cryptocurrency (Huynh, 2020a, 2020b) and the influence of fear sentiment on cryptocurrency (Burggraf et al., 2020a, 2020b). Other studies have also revealed the role of Twitter on the cryptocurrency market (Kraaijeveld & De Smedt, 2020; Naeem et al., 2020a, 2020b; Öztürk & Bilgiç, 2021). Using textual analysis, our study contributes fresh evidence of the influence of Musk’s sentiments on this market. It is reasonable to conclude that this market is relatively sensitive to news, particularly tweets posted by influential individuals.

### Predictive power of Elon Musk’s sentiments on Bitcoin returns

In this section, we employ regressions to examine whether Musk’s sentiments could predict Bitcoin returns. Our model specifications are written as follows.

$$ {\text{Bitcoin}}_{{\text{t}}} = \beta_{0} + \mathop \sum \limits_{{{\text{i}} = 0}}^{{\text{p}}} {\updelta }_{{\text{i}}} {\text{Bitcoin}}_{{{\text{t}} - {\text{p}}}} + \mathop \sum \limits_{{{\text{i}} = 0}}^{{\text{p}}} \phi_{{\text{i}}} {\text{Negsent}}_{{{\text{t}} - {\text{p}}}} + \mathop \sum \limits_{{{\text{i}} = 0}}^{{\text{p}}} \varpi_{{\text{i}}} {\text{Possent}}_{{{\text{t}} - {\text{p}}}} + \mathop \sum \limits_{{{\text{t}} = 0}} \psi {\text{Control}} + \varepsilon_{{\text{t}}} { }\left( {{\text{Model }}1} \right) $$

Here, ${Bitcoin}_{t}$ represents Bitcoin returns at day t. In our main model, we focus on the lagged terms of Bitcoin returns, and Musk’s negative (*Negsent*) and positive (*Possent*) sentiments. ${\beta }_{0}$,$\phi$, and $\varpi$ denote a constant term, and coefficient parameters for Negsent and Possent, respectively. The last term, $Control$, is a set of control variables with parameters ($\psi$). Here, ${\upvarepsilon }_{\mathrm{t}}$ is an error term. More importantly, p is the estimated optimal lag.^1^ For control variables, we employed the solid and sound factors to control the movement of Bitcoin returns. In particular, the natural logarithm of the ratio of Gold and Platinum prices reported by Huynh et al. (2020a, 2020b) was found to be a predictive factor for Bitcoin returns. This indicator represents the aggregated market risk. Alternative investment indicators, such as the global bond index and CDSs, are included. Furthermore, a recent study by Bekaert et al. (2019) emphasized the price of risk, proxied by the risk aversion index. Table 2 demonstrates the predictive power of Elon Musk’s sentiments on Bitcoin returns.

**Table 2:** Predictive power of Elon Musk sentiments on Bitcoin returns

Variables	Bitcoin_(t)_	Bitcoin_(t)_
Bitcoin_(t−1)_	− 0.157 [− 0.942]	− 0.328*** [− 2.669]
Bitcoin_(t−2)_	0.593*** [2.639]	0.280* [1.730]
Bitcoin_(t−3)_	− 0.292* [− 1.878]	− 0.100 [− 0.769]
Negsent_(t−1)_	1.841*** [2.714]	1.224** [2.479]
Negsent_(t−2)_	0.032 [0.042]	0.045 [0.090]
Negsent_(t−3)_	0.231 [0.353]	1.498*** [2.821]
Possent_(t−1)_	1.282* [1.756]	1.213** [2.230]
Possent_(t−2)_	1.474 [1.231]	2.577*** [2.815]
Possent_(t−3)_	− 1.688** [− 2.560]	− 1.685*** [− 3.444]
Constant	− 0.026 [− 0.996]	− 0.134*** [− 3.581]
R-squared	0.613	0.848
F-stat	38.128***	128.314***
Control variables	No	Yes

We employed the Vector Auto-Regression (VAR) approach for estimating the short-term relationship. Our t-statistics are reported in parentheses. Control variables consist of the natural logarithm of a ratio between Gold and Platinum (GP) from the study of Huynh et al. (2020a, 2020b); the index of Credit Default Swaps (CDS); the FTSE bond index for clean price, and Risk Aversion Index as the price of risk by Bekaert et al. (2019). We obtained the residual from model for normality test (*p* = 0.19 > 0.10) and ADF test (t-stat =  − 4.576, *p* < 0.01)

*< 0.1; **< 0.05; ***< 0.01

Interestingly, negative sentiment positively predicts Bitcoin returns when the lagged terms of a single period and three periods are significant at a 1% level. The positive tone in one and two lagged periods could positively predict Bitcoin returns at least a 5% significance level. However, we observe reversal effects in three lagged terms (negative coefficient at 1% significance level). Our study dives deeper in the literature that market attention toward the influencer’s tones. Huynh (2021) investigated the role of a risk premium under uncertainties and found that Trump’s tweets could predict Bitcoin markets. Our study also differs from the event study conducted by Ante (2021). Ante’s study presented empirical evidence that Elon Musk’s tweets could move cryptocurrency markets. In contrast, our study constructed a new indicator, i.e., influencer tone as aggregated market risk. We found that when the world’s richest person expresses both pessimistic and optimistic attitudes, investors are likely to move their capital to the referenced market as a safe-haven with higher risk premium requirements. The Granger causality Wald test also indicated a causal relationship between Musk’s sentiments and Bitcoin returns. We reject the hypothesis that negative attitudes, positive attitudes could not drive Bitcoin returns (${\chi }^{2}$ = 12.497 and 23.935, respectively; *p* < 0.01). It implies that our predictive power involves both correlation and causality. Obviously, the role of influencers on investor attention in cryptocurrency markets should be considered. While it may be intuitive to expect that the view of the richest person in the world could drive Bitcoin returns, our study provides evidence that the causal relationship can be pronounced.

Our finding are consistent with previous studies based on a theoretical framework and demonstrate that changes in Musk’s attitudes could require a higher discounted premium (Huynh, 2021; Schwert, 1981; Wagner et al., 2018). Therefore, we can see that, by requiring a higher discounted premium, investor behavior is irrational. In other words, both Musk’s optimistic and pessimistic views cause feelings of happiness. Thus, Elon Musk's tone could hype investors’ feelings. More noticeably, investors demonstrate reversal effects, i.e., they tend to require a greater premium when Musk initially expresses an optimistic sentiment. Then, investors might think that they have overreacted; then, investors will likely change their decisions and take corrective action.

### Predictive Power of Elon Musk’s Sentiments on Bitcoin Volatility

**Fig. 2:**
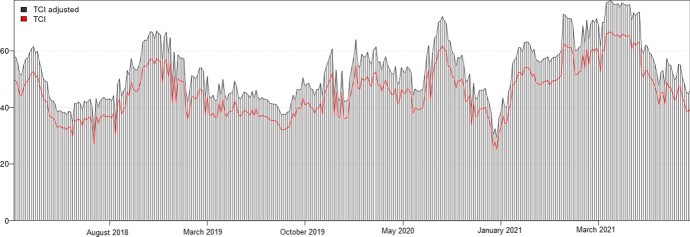
Total dynamic connectedness between Bitcoin volatility and Elon Musk’s sentiments. *Notes*: The total dynamic connectedness between Bitcoin volatility and two tones (positive and negative attitudes) by using the dynamic conditional correlation–generalized autoregressive conditional heteroscedasticity (DCC-GARCH) models. Our research period is from December 2017 to May 2021. TCI denotes the total connectedness index. The figure practically implies that the connectedness between Elon Musk’s sentiment and Bitcoin volatility

We used the T-GARCH model to estimate Bitcoin volatility. The T-GARCH model is the conventional GARCH with the asymmetric effects of news incorporated. The model specifications of T-GARCH with conditional variance can be expressed as:

13 $$ h_{t} = \delta + \alpha_{1} e_{t - 1}^{2} + \gamma d_{t - 1} e_{t - 1}^{2} + \beta_{1} h_{t - 1} $$

14 $$ d_{t} = \left\{ {\begin{array}{*{20}c} {\begin{array}{*{20}c} 1 & {e_{t} < 0 \left( {\text{bad news}} \right)} \\ \end{array} } \\ {\begin{array}{*{20}c} 0 & {e_{t} \ge 0 \left( {\text{good news}} \right)} \\ \end{array} } \\ \end{array} } \right. $$

where ${h}_{t}$ denotes the squared error over the preceding period. Note this parameter should be greater than zero. Here, ${e}_{t}^{2}$ denotes the square of the estimated residuals at time t, and d denotes two news scenarios. Parameters $\delta$, $\alpha$, $\gamma$, and $\beta$ represent constant terms, squared residual without news, squared residual with a binary option regarding news, and the autoregressive term, respectively. Figure 2 captures the dynamic connectedness between Elon Musk’s sentiments and Bitcoin volatility with all control variables. As expected, the highest number of tweets occurred since 2021 because Elon Musk is likely to tweet frequently after he owned a large number of cryptocurrencies.

Table 3 summarizes how Musk’s tone could predict Bitcoin volatility. In general, all coefficients of Musk’s sentiments are insignificant, which means that there is no relationship between the influencer tone and Bitcoin volatility. In addition, we did not find any evidence of a causal relationship between sentiments and Bitcoin volatility. Accordingly, the ${\chi }^{2}$ for negative and positive sentiment are 0.034 and 0.297 (*p* > 0.1), respectively with control variables. These values are 0.066 and 0.452 (*p* > 0.1) without control variables.

**Table 3:** Predictive power of Elon Musk sentiments on Bitcoin volatility

Variables	Volatility_(t)_	Volatility_(t)_
Volatility_(t−1)_	1.466*** [30.767]	1.476*** [29.878]
Negsent_(t−1)_	− 0.001 [− 0.258]	− 0.001 [− 0.187]
Possent_(t−1)_	− 0.002 [− 0.673]	− 0.002 [− 0.545]
Constant	− 0.001*** [− 9.262]	− 0.001*** [− 5.653]
R-squared	0.8715	0.8729
F-stat	949.304***	961.567***
Control variables	No	Yes

We employed the Vector Auto-Regression (VAR) approach for estimating the short-term relationship. Our t-statistics are reported in parentheses. Control variables consist of the natural logarithm of a ratio between Gold and Platinum (GP) from the study of Huynh et al. (2020a, 2020b); the index of Credit Default Swaps (CDS); the FTSE bond index for clean price, and Risk Aversion Index as the price of risk by Bekaert et al. (2019). The optimal lag (one term) was based on the consistent criteria of AIC, HQIC, and SBIC. The Bitcoin volatility was estimated by using Threshold GARCH and the results are available upon request. We also obtained the residual in the aforementioned model and performed the normality test and stationary. The results indicated that the residual having non-normal distribution (*p* < 0.05) but stationary at 1% significance level (t-stat =  − 43.084, *p* < 0.01)

*< 0.1; **< 0.05; ***< 0.01

Contradicting a previous finding (Shen et al., 2019), considering the variance, we did not find any evidence that Musk’s Twitter posts were associated with Bitcoin volatility. This indicates that the market's reactions might have a short horizon (Ante, 2021), e.g., for 30 min or several hours. Another explanation is that, different from Trump’s tweets, the Elon Musk effect could not be seen as aggregate market risk. Therefore, investors want to earn abnormal returns and have the market return to equilibrium in the following trading periods. Shen et al. (2019) emphasized the role of realized volatility while our study employed T-GARCH to handle leverage effects (Zakoian, 1994). Therefore, our study also provides new empirical evidence that differences in volatility calculations could cause analysis results to diverge.

### Robustness Check

We replaced Bitcoin returns with Ethereum returns from the Binance exchange to evaluate the robustness of our proposed approach. Table 4 shows the predictive power of Elon Musk sentiments on Ethereum returns with optimal lagged terms. We found that both optimistic and pessimistic sentiments could predict the Ethereum returns at least a 5% significance level. Moreover, Granger causality also confirmed the validity of these factors on the alternative cryptocurrency (Ether) (*p* < 0.05). More noticeably, similar to the Bitcoin case, positive sentiments also demonstrated reversal effects. Therefore, our study also confirms the interchangeable features of Bitcoin and Ethereum when reacting to news. Our findings are in-line with the robustness methodology of when FEARS can be a predictive power on both cryptocurrencies. More importantly, Huynh (2021) also found that Donald Trump’s sentiments may drive Bitcoin and Ethereum returns. The effects are likely to be stronger during the pandemic. Motivated by the story of COVID-19 pandemic of Trump’s tone (Huynh, 2021), we decided to use Tesla returns to control Elon Musk’s sentiments. Our results remain robust and are even stronger than the baseline models. We also controlled for alternative equity indices that might be affected by Elon Musk’s sentiments, such as the NASDAQ artificial intelligence and robotics stock index (Huynh et al., 2020a, 2020b).^2^

**Table 4:** Predictive power of Elon Musk sentiments on Ethereum returns

Variables	Ethereum_(t)_	Ethereum_(t)_
Ethereum_(t−1)_	− 0.221 [− 1.608]	− 0.486*** [− 5.733]
Ethereum_(t−2)_	0.341* [1.917]	− 0.048 [− 0.498]
Ethereum_(t−3)_	− 0.166 [− 1.305]	0.053 [0.708]
Negsent_(t−1)_	1.959*** [2.638]	0.549 [1.280]
Negsent_(t−2)_	− 0.254 [− 0.305]	− 0.457 [− 1.152]
Negsent_(t−3)_	− 0.193 [− 0.280]	1.086** [2.573]
Possent_(t−1)_	0.027 [0.034]	1.112** [2.325]
Possent_(t−2)_	0.372 [0.288]	1.654** [2.373]
Possent_(t−3)_	− 1.342* [− 1.855]	− 0.798** [− 2.034]
Constant	0.014 [0.493]	− 0.140*** [− 4.720]
R-squared	0.533	0.899
F-stat	27.418***	214.658***
Control variables	No	Yes

We employed the Vector Auto-Regression (VAR) approach for estimating the short-term relationship. Our t-statistics are reported in parentheses. Control variables consist of the natural logarithm of a ratio between Gold and Platinum (GP) from the study of Huynh et al. (2020); the index of Credit Default Swaps (CDS); the FTSE bond index for clean price, and Risk Aversion Index as the price of risk by Bekaert et al. (2019)

*< 0.1; **< 0.05; ***< 0.01

## Conclusion

This paper sheds new light on the potential relationship between Elon Musk’s Twitter feed and Bitcoin markets. Tesla’s CEO tends to use an optimistic tone when communicating with users on his Twitter account. This study also found that the effects of Musk’s attitudes could drive Bitcoin returns on the short trading days. More importantly, the reversal effects are pronounced for optimistic views with one lagged trading day. However, we did not find any evidence for impacts of Elon Musk’s Twitter feed on Bitcoin volatility. This implies quick and efficient adjustments from investors after gaining the *advantageous information* from the frangibility of Bitcoin markets. After controlling rigorous variables, we found that the effects are persistent with the time-varying analyses based on the DCC-GARCH model. Our results remain robust when using an alternative cryptocurrency, which implies that Elon Musk’s sentiments can be widely applied to predict risk premiums in cryptocurrency markets.

Our study has three main implications. First, we argue that the Bitcoin market can be driven by external information, and that cryptocurrency prices can be easily manipulated by one specific Twitter account. This impedes transparent trading schemes and potentially creates unnecessary market makers. Second, investors, hedge funds, and market participants should pay attention to Elon Musk’s social media content and choose appropriate strategies to avoid any sudden changes in the market. Third, the economic and computational model should be incorporated into textual analysis to obtain better forecasting. Incorporating news sentiment in the model could improve prediction quality (Chen & Lux, 2018; Xu & Hsu, 2021).

Since this study uses daily data to examine the impacts of Elon Musk’s tone, future studies could investigate intra-day data, which indicates market structure changes after releasing information. Further research directions include monitoring and forecasting cryptocurrency returns and volatility by combining different social media information, e.g., information released by the CEO of the Binance exchange or Vitalik Buterin, the creator of Etheruem.

## Figures and Tables

**Fig. 3:**
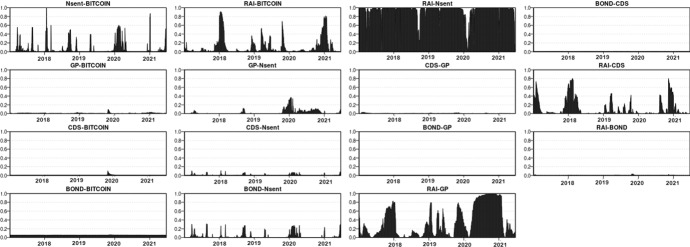
Dynamic pairwise of all variables with Elon Musk’s negative sentiments. *Notes*: These figures summarized the net dynamic pairwise between Elon Musk’s negative sentiments and all variables. In which, Nsent is the Elon Musk’s negative sentiment. Bitcoin, GP, CDS, Bond, and RAI are the Bitcoin return, the nature logarithm of Gold and Platinum, Credit Default Swap, the FTSE global bond index, and Risk Aversion Index, respectively. The pair ‘A-B’ means ‘A’ as the sending position and ‘B’ as the receiving position

**Fig. 4:**
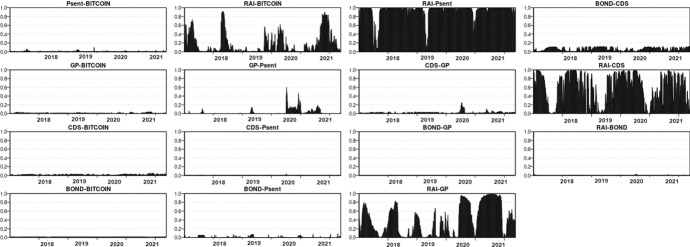
Dynamic pairwise of all variables with Elon Musk’s positive sentiments. *Notes*: These figures summarized the net dynamic pairwise between Elon Musk’s positive sentiments and all variables. In which, Psent is the Elon Musk’s positive sentiment. Bitcoin, GP, CDS, Bond, and RAI are the Bitcoin return, the nature logarithm of Gold and Platinum, Credit

**Fig. 5:**
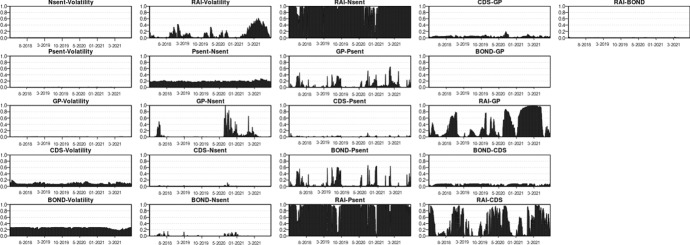
Dynamic pairwise of all variables (included volatility) with Elon Musk’s sentiments. *Notes*: These figures summarized the net dynamic pairwise between Elon Musk’s sentiments and all variables, including Bitcoin volatility. In which, Nsent and Psent are the Elon Musk’s negative positive sentiment, respectively. Volatility, GP, CDS, Bond, and RAI are the Bitcoin volatility, the nature logarithm of Gold and Platinum, Credit Default Swap, the FTSE global bond index, and Risk Aversion Index, respectively. The pair ‘A-B’ means ‘A’ as the sending position and ‘B’ as the receiving position

## Data Availability

The data will be available upon request.

## Appendix

See Tables 5 and 6, 7 and Figs. 3, 4 and 5.

**Table 5:** Dynamic connectedness between Elon Musk’s negative sentiments on Bitcoin returns

	BITCOIN	NEGSENT	GP	CDS	BOND	RAI	FROM
BITCOIN	**86.42**	0.00	0.51	0.13	2.64	10.31	13.58
NEGSENT	4.05	**2.02**	0.79	0.41	1.79	90.94	97.98
GP	0.02	0.00	**65.46**	0.01	0.01	34.5	34.54
CDS	0.58	0.00	0.38	**87.36**	0.63	11.05	12.64
BOND	2.69	0.00	0.2	0.14	**96.82**	0.15	3.18
RAI	0.00	0.00	0.35	0.00	0.00	**99.65**	0.35
Contribution TO others	7.34	0.00	2.23	0.68	5.07	146.95	**162.27**
NET directional connectedness	− 6.24	− 97.98	− 32.31	− 11.95	1.89	146.59	TCI
NPDC transmitter	2.00	5.00	1.00	4.00	3.00	0.00	**27.04**

Numbers summarized are variance decompositions based on 100-day-ahead forecasts from the DCC‐GARCH models from Gabauer (2020)

**Table 6:** Dynamic connectedness between Elon Musk’s positive sentiments on Bitcoin returns

	BITCOIN	POSSENT	GP	CDS	BOND	RAI	FROM
BITCOIN	**81.54**	0.00	0.74	0.01	0.95	16.76	18.46
POSSENT	0.28	**7.95**	0.70	0.03	0.61	90.43	92.05
GP	0.02	0.00	**60.99**	0.00	0.00	38.99	39.01
CDS	1.13	0.00	0.96	**37.47**	4.89	55.55	62.53
BOND	0.32	0.00	0.01	0.02	**99.52**	0.12	0.48
RAI	0.00	0.00	0.26	0.00	0.00	**99.74**	0.26
Contribution TO others	1.75	0.00	2.67	0.07	6.45	201.85	**212.79**
NET directional connectedness	− 16.71	− 92.05	− 36.34	− 62.46	5.97	201.59	TCI
NPDC transmitter	3.00	5.00	1.00	4.00	2.00	0.00	**35.46**

Numbers summarized are variance decompositions based on 100-day-ahead forecasts from the DCC‐GARCH models from Gabauer (2020)

**Table 7:** Dynamic connectedness between Elon Musk’s sentiments on Bitcoin volatility

	BITCOIN	NEGSENT	POSSENT	GP	CDS	BOND	RAI	FROM
BITCOIN	**60.06**	0.00	0.01	0.22	4.73	23.99	10.98	39.94
NEGSENT	0.01	**3.58**	0.40	2.12	0.10	0.64	93.14	96.42
POSSENT	0.05	0.18	**1.93**	2.81	0.19	5.76	89.08	98.07
GP	0.00	0.00	0.00	**63.89**	0.00	0.02	36.09	36.11
CDS	0.21	0.00	0.00	2.54	**53.21**	4.79	39.25	46.79
BOND	0.01	0.00	0.00	0.27	0.06	**99.55**	0.11	0.45
RAI	0.00	0.00	0.00	0.24	0.00	0.00	**99.76**	0.24
Contribution TO others	0.29	0.18	0.42	8.20	5.08	35.20	268.66	**318.02**
NET directional connectedness	− 39.65	− 96.24	− 97.65	− 27.91	− 41.71	34.75	268.42	TCI
NPDC transmitter	4.00	6.00	5.00	1.00	3.00	2.00	0.00	45.43

Numbers summarized are variance decompositions based on 100-day-ahead forecasts from the DCC‐GARCH models from Gabauer (2020). Default Swap, the FTSE global bond index, and Risk Aversion Index, respectively. The pair ‘A-B’ means ‘A’ as the sending position and ‘B’ as the receiving position

## Footnotes

1 Our selection-order criteria based on AIC, HQIC, and SBIC is three.

2 Our results are available upon request.

## Code Availability (Software Application or Custom Code)

The code will be available upon request.
